# Environmental Impact of Dietary Choices: Role of the Mediterranean and Other Dietary Patterns in an Italian Cohort

**DOI:** 10.3390/ijerph17051468

**Published:** 2020-02-25

**Authors:** Giuseppe Grosso, Ujué Fresán, Maira Bes-Rastrollo, Stefano Marventano, Fabio Galvano

**Affiliations:** 1 Department of Biomedical and Biotechnological Sciences, University of Catania, 95123 Catania, Italy; fgalvano@unict.it; 2 CIBER-ESP, 28029 Madrid, Spain; ufresan@llu.edu; 3 Instituto de Salud Pública y Laboral de Navarra, 31003 Pamplona, Spain; 4 Department of Preventive Medicine and Public Health, University of Navarra, 31008 Pamplona, Spain; mbes@unav.es; 5 IdiSNA, Navarra’s Health Research Institute, 31008 Pamplona, Spain; 6 CIBERobn, Instituto de Salud Carlos III, 28029 Madrid, Spain; 7 Department of Childhood and Adolescent, AUSL Romagna, Rimini Women’s Health, 47921 Rimini, Italy; stefanomarv@gmail.com

**Keywords:** sustainability, Mediterranean diet, DASH, Nordic diet, diet quality, dietary pattern, cohort, greenhouse gas emission, energy use, water use

## Abstract

*Background*: Current scientific literature suggests healthy dietary patterns may have less environmental impact than current consumption patterns, but most of the studies rely on theoretical modeling. The aim of this study was to assess the impact on resources (land, water, and energy) use and greenhouse gas (GHG) emissions of healthy dietary patterns in a sample of Italian adults. Methods: Participants (*n* = 1806) were recruited through random sampling in the city of Catania, southern Italy. Dietary consumption was assessed through a validated food frequency questionnaire (FFQ); dietary patterns were calculated through dietary scores. The specific environmental footprints of food item production/processing were obtained from various available life-cycle assessments; a sustainability score was created based on the impact of the four environmental components calculated. Results: The contribution of major food groups to the environmental footprint showed that animal products (dairy, egg, meat, and fish) represented more than half of the impact on GHG emissions and energy requirements; meat products were the stronger contributors to GHG emissions and water use, while dairy products to energy use, and cereals to land use. All patterns investigated, with the exception of the Dietary Approach to Stop Hypertension (DASH), were linearly associated with the sustainability score. Among the components, higher adherence to the Mediterranean diet and Alternate Diet Quality Index (AHEI) was associated with lower GHG emissions, dietary quality index-international (DQI-I) with land use, while Nordic diet with land and water use. Conclusions: In conclusion, the adoption of healthy dietary patterns involves less use of natural resources and GHG emissions, representing eco-friendlier options in Italian adults.

## 1. Introduction 

Over the last few years, a great effort has been made to bring to global attention to the impact of human activities on planetary health. Among others, food production is considered a major driver of environmental change: it has been estimated that food production is responsible for about one-third of global greenhouse gas (GHG) emissions and two-thirds of freshwater use; agriculture and fishery represent an important burden for global land and marine systems; climate change, chemical pollution, and unsustainable harvesting play a considerable role in habitat ecosystems fragmentation and, ultimately, on biodiversity loss [1]. Importantly, dietary choices link environmental sustainability and human health [2]. In fact, the global shift toward unhealthy dietary habits represents a major contributor to the burden of obesity and dietary-related non-communicable diseases [3]. Thus, besides their environmental impact, current dietary trajectories are strongly affecting the mortality risk for a number of non-communicable diseases, including cardiovascular disease (CVD), type-2 diabetes, and certain cancers, accounting for 11 million deaths in 2017 [4]. If current trends remain unchecked, food production will be the major contributor to global GHG emissions and global land clearing as well as the leading behavioral risk factor for premature death worldwide [5].

The UN Sustainable Development Goals (SDGs) agenda includes various points involving nutrition-related goals, including ending hunger and all forms of malnutrition, good health and wellbeing for all, together with a low environmental impact and sustainable approach to preserve the world health [6]. A definition for sustainable diet has been considered as “with low environmental impacts which contribute to food and nutrition security and to healthy life for present and future generations” with specific features, such as “protective and respectful of biodiversity and ecosystems, culturally acceptable, accessible, economically fair and affordable; nutritionally adequate, safe and healthy; while optimizing natural and human resources” [7]. Global modeling analysis with country-level detail showed that following environmental objectives by replacing animal-source foods with plant-based ones would be particularly effective in high-income countries for improving nutrient levels, lowering premature mortality, and reducing some environmental impacts [8]. Recently, the latest report from the EAT-Lancet commission of global experts concluded that a sustainable dietary pattern would optimally include primarily plant-derived protein sources, fats mostly from unsaturated plant sources, carbohydrates from whole grains, fruit, and vegetable; moderate dairy and egg consumption would be optional [9]. It is crucial to raise awareness among policy makers, the food supply chain, and the individual consumers in order to lead to a transformation of the global food system. Major emphasis on dietary patterns, rather than individual foods, is warranted to provide a more comprehensive evaluation of the whole diet in relation to health or environment. Although not explicitly provided in the definition of a sustainable diet, it is noteworthy to emphasize that consumption of locally produced foods may positively affect the environmental impact of dietary habits. In this context, the Mediterranean diet has been considered a valuable example of healthy and sustainable dietary pattern [10]: the Mediterranean diet refers to the traditional dietary pattern adopted by southern Italian population in the 1960s [11] that only recently has been declared as intangible cultural heritage of humanity by UNESCO due to its cultural value as part of the cultural heritage of a population [12,13], which provides, in addition, a number of health benefits [14,15]. Importantly, the Mediterranean dietary pattern has been investigated to be potentially applied or modified to be adopted globally [16,17]. Modeling analyses have been conducted [18], but empirical investigation on the agreement between dietary patterns and environmental impact are scarce. This study aimed to investigate whether higher adherence to healthy dietary patterns may be associated with less environmental impact.

## 2. Materials and Methods

### 2.1. Study Population

Study participants belong to the Mediterranean Healthy Eating, Aging, and Lifestyles (MEAL) study, a cross-sectional study investigating dietary and lifestyle habits of adults living in the urban Mediterranean area [19]. A random sample of men and women aged 18 or more was recruited during 2014–2015 among the registered data of local general practitioners in the urban area of Catania, southern Italy. Participants randomly selected for recruitment were stratified by sex and 10-year age groups (pregnant women were not considered). The theoretical sample size was set at 1500 individuals to provide a specific relative precision of 5% (Type I error, 0.05; Type II error, 0.10), taking into account an anticipated 70% participation rate. Out of 2405 individuals invited, the final sample size was 2044 participants (response rate of 85%). All the study procedures were carried out in accordance with the Declaration of Helsinki (1989) of the World Medical Association. Participants provided written informed consent, and the study protocol was approved by the ethics committee of the referent health authority (number 802/23 December 2014).

### 2.2. Data Collection

Demographic (age, sex, educational, and occupational level) and lifestyle characteristics (i.e., physical activity, smoking, and drinking habits) were collected. Educational level was categorized as: (i) low (primary/secondary), (ii) medium (high school), and (iii) high (university). Occupational level was classified as: (i) unemployed, (ii) low (unskilled workers), (iii) medium (partially skilled workers), and (iv) high (skilled workers). Physical activity level was assessed using the International Physical Activity Questionnaires (IPAQ) [20], which comprised a set of questionnaires (5 domains) on time spent being physically active in the last 7 days that allow categorizing physical activity as: (i) low, (ii) moderate, and (iii) high. Smoking status was classified as: (i) non-smoker, (ii) ex-smoker, and (iii) current smoker. Alcohol consumption was categorized as (i) none, (ii) moderate drinker (0.1–12 g/d) and (iii) regular drinker (>12 g/d). Anthropometric measurements were performed according to standardized methods [21]. Height was measured to the nearest 0.5 cm without shoes, with the back square against the wall tape, eyes looking straight ahead, with a right-angle triangle resting on the scalp and against the wall. Body mass index (BMI) was calculated, and patients were categorized as under/normal weight (BMI < 25 kg/m^2^), overweight (BMI 25 to 29.9 kg/m^2^), and obese (BMI ≥ 30 kg/m^2^) [22].

### 2.3. Dietary Assessment and Dietary Patterns

Dietary data were collected using a long and a short version of a food frequency questionnaire (FFQ) validated for the Sicilian population [23,24]. The long FFQ consisted of 110 food and drink items representative of the diet during the last 6 months. Participants of the study were asked how often, on average, they had consumed foods and drinks included in the FFQ, with nine responses ranging from “never” to “4–5 times per day”. Intake of food items characterized by seasonality referred to consumption during the period in which the food was available and then adjusted by its proportional intake over one year. FFQ reporting unreliable extreme dietary intakes (*n* = 107) or missing food items needed for calculation of the environmental variables (*n* = 131) were excluded, leaving a total of 1806 individuals included in the analyses for the present study.

Various dietary patterns have been calculated on the basis of a priori defined criteria according to the scientific literature. A Mediterranean diet adherence score was calculated with a maximum of 9 points, assigning 1 point whether daily servings of vegetables, legumes, fruits and nuts, cereals, and fish, and the ratio of grams of MFA to grams of SFA were equal to or greater than the sex-specific median intake of the study population; daily servings of meat (including red meat, poultry, organ meats, and processed meats) and dairy foods were less than the sex-specific median intake of the study population; and alcohol intake was 10–50 g/d for men and 5–25 g/d for women [25]. Adherence to the Dietary Approaches to Stop Hypertension (DASH) was calculated with a 9-point score assigned to men and women based on the consumption of the recommended number of daily servings from each of the 6 main food groups (whole grain, vegetable, fruits, dairy products, meat, and nuts/seeds/legumes); men and women also received 1 point if their average daily consumption of saturated fat was less than the recommended 5% of total energy intake, if their added sugar intake was <3% of total energy intake, and if their alcohol intake was at or below the recommended 2 drinks/d for men and 1 drink/d for women [26]. The Nordic diet score was calculated based on a previous study [27]: the consumption of 9 components considered characteristics of this dietary pattern (whole grain and rye bread, berries, apples and pears, fish, cabbage and cruciferous vegetables, root vegetables, low-fat dairy products, potatoes, and vegetable fats excluding olive oil) was categorized in sex-specific tertiles of intake and assigned a score of 0 to 2 points according to the first, second, and third tertiles, respectively, ranging from a total of 0 to 18 points. Two additional indices were used to measure diet quality. The Alternate Diet Quality Index (AHEI) [28] included 8 components (vegetables, fruits, nuts, ratio of white to red meat, cereal fiber, *trans* fat, alcohol, ratio of polyunsaturated to saturated fatty acids) each contributing 0–10 points to the total score: a score of 10 indicates that the recommendations were fully met, whereas a score of 0 represents the least healthy dietary behavior and intermediate intakes were scored proportionately between 0 and 10; the use of a multivitamin was a ninth item accounted as dichotomous, contributing either 2.5 points (for nonuse) or 7.5 points (for use); all component scores were summed to obtain a total AHEI score ranging from 2.5 (worst) to 87.5 (best). The dietary quality index-international (DQI-I) explores four major domains considered necessary to a healthy diet, such as variety, adequacy, moderation, and overall balance. The DQI-I incorporates an amount of both nutrients and foods in the assessment, providing a means with which to better describe the diversity of consumption from country to country. The basic rationale for the construction of the DQI-I has been described elsewhere [29]. The main four domains were evaluated as follows: (i) Variety was assessed by considering inclusion of serving per day of five main food groups (meat/poultry/fish/egg, dairy/beans, grains, fruits, and vegetables), with consumption of at least one of each food group indicating maximum overall variety score; (ii) adequacy was assessed considering minimum intakes of key dietary elements, such as fruit, vegetables, grains, and fiber, dependent on energy intake. Cut off values of 7118 kJ (1700 kcal), 9211 kJ (2200 kcal) and 11,304 kJ (2700 kcal) were used to define better diet quality with daily consumption of two, three, and four portions of fruit; three, four, and five portions of vegetable; >6, >9 and >11 portions of grains; and >20, >25 and >30 g of fiber, respectively. Proportion of total energy from protein >10% was considered adequate. Cut-off values for iron, calcium, and vitamin C intake were derived from the recommended daily intakes for Italian adults [30,31]; (iii) moderation was assessed considering maximum intakes of food and nutrients that may need restriction; (iv) overall balance indicated the proportion of energy sources and fatty acid composition. The scoring system consisted in an individual score for each of the main categories and an overall summary of all points ranging from 0 to 100 (0 being the poorest and 100 being the highest possible score).

### 2.4. Environmental Variables

The environmental impact of the food items collected in the FFQ was based on a dataset previously described [32]. Briefly, the assessment of the natural resources (i.e., water, land, and energy) use and GHG emissions of each food product had been assessed based on secondary data, collected from several scientific sources (please see Supplementary Materials) [32]. The system boundaries had been food production and processing (except for land use, which included crops and livestock production, but not land related to food processing). Only conventional agriculture processes had been considered.

The specific value that a serving of each item had in relation to resource use or GHG emissions was multiplied by the number of servings of that item consumed per day by each participant, obtaining the daily impact of each food item on the four environmental domains investigated. We summed the values of all food items, obtaining the impact on the water, land and energy use, and GHG emissions of the daily diet of each participant. Finally, a sustainability score was calculated by assigning 0 or 1 point to each of these four components using the sex-specific medians as the cut-offs (0 for upper values and 1 for lower ones), ranging from a total of 0 to 4 points, with higher scores indicating less environmental impact.

### 2.5. Statistical Analysis

Background categorical variables are presented as frequencies of occurrence and percentages differences according to sustainability score groups (categorized based on the points 0–1, 2–3, and 4) were tested using the Chi-squared test. Dietary intakes are presented as mean and standard deviations (SDs); differences between groups were tested with Student’s t-test. Linear regression analyses adjusted for background variables were performed to assess the association between dietary patterns investigated and the sustainability score and its individual components. All reported *p* values were based on two-sided tests and compared to a significance level of 5%. SPSS 17 (SPSS Inc., Chicago, IL, USA) software was used for all the statistical analysis.

## 3. Results

The background characteristics of the study sample distributed according to sustainability score groups are presented in Table 1. None of the variables investigated were distributed differently among sustainability score groups besides physical activity levels, despite the fact that no clear trend can be observed (Table 1).

The distribution of micro-, macro-nutrients, and food groups according to sustainability score groups is presented in Table 2. As expected, most of the food groups providing lower environmental impact (i.e., cereals, fruit, and legumes) were more consumed with increasing sustainability score; moreover, some food groups with higher environmental impact (i.e., meat and fish) had similar trends of intake; in contrast, foods with average environmental impact (i.e., olive oil and egg) had an inverse trend of consumption (more consumed with decreasing sustainability score group; Table 2). Concerning macro-nutrients, significant trends over higher intake with an increasing sustainability score were found for saturated, mono-, and polyunsaturated fatty acids (including omega-3 and cholesterol), protein, carbohydrate, and fiber (Table 2). Among micro-nutrients, similar results were found for vitamin C, E, and B12 (Table 2).

**Figure 1:**
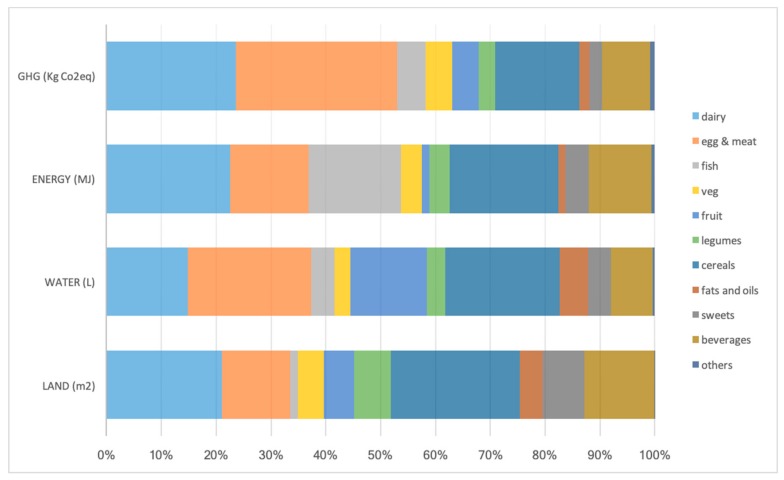
Contribution of major food groups to the four environmental factors.

When considering the contribution of major food groups to the components of the sustainability score, animal products (dairy, egg, meat, and fish) represented more than half of the impact on GHG emissions and energy requirements; meat products were the stronger contributors to GHG emissions and water, while dairy products to energy, and cereals to land use (Figure 1).

Table 3 shows the association between various dietary patterns and the sustainability score and its components. All patterns investigated, with the exception of the DASH, were linearly associated with the sustainability score. Among the components, higher adherence to the Mediterranean diet and AHEI was associated with lower GHG emissions, DQI-I with land use, while Nordic diet with land and water use.

## 4. Discussion

In this study, the Mediterranean diet and other healthy dietary patterns have been associated with environmental impacts, and higher adherence to them resulted in less environmental impact in a cohort of Italian adults. Most of the existing evidence relies on theoretical models on the relation between dietary patterns and environmental sustainability. A report systematically reviewing the evidence from modeling studies on changes in GHG emissions, land use, and water use, showed that shifting from current to environmentally sustainable dietary patterns would exert an impact in all outcomes investigated; among the dietary patterns investigated, meat-free diets showed the lowest environmental impact together with a Mediterranean-type diet [18]. Modeling studies considering Mediterranean countries led to similar conclusions [33]. Concerning the southern Italian population, we previously reported there is evidence of a certain abandonment of traditional dietary patterns (including the Mediterranean diet) toward Westernized ones [34,35,36]: the reasons for such trends have been attributed to economical constraints of the population or rise in prices in healthy products compared to the globalized market [37,38]. As a result, a higher adherence to the Mediterranean diet would produce a lower environmental impact than the current food consumption, and the monthly expenditure would be slightly higher in the overall budget compared to the current expenditure allocated to food by the Italian population [39]. These considerations are in line with the results from the few reports using empirical data from cohort studies. A previous study conducted on the SUN cohort (including Spanish individuals) sharing similar methodology showed that better adherence to the Mediterranean diet was associated with lower land use, water consumption, energy consumption, and GHG emissions in a linear manner [32]. In a prospective observation, the findings were confirmed, as both a Mediterranean and a pro-vegetarian dietary pattern would exert the best health benefits and the lowest environmental effects; notably, the Western pattern was the most affordable while the Mediterranean pattern was the most expensive choice [40]. Also, data from the European Prospective Investigation into Cancer and Nutrition—Netherlands (EPIC-NL) cohort showed that the WHO and Dutch dietary guidelines will lower the risk of all-cause mortality and moderately lower the environmental impact, while the DASH diet, despite leading to similar health outcomes, was associated with higher GHG emissions due to high dairy product consumption in the Netherlands [41]. Another study conducted on Italian adults showed that omnivorous dietary choices generated worse carbon, water, and ecological footprints than other plant-based diets, while no differences were found for the environmental impacts of ovo-lacto-vegetarians and vegans [42], which also had dietary habits closer to the main features characterizing the Mediterranean pattern [43].

Previous consensus papers addressed the Mediterranean diet as a healthy and sustainable dietary pattern [44]. The scientific literature provides convincing evidence of the beneficial effect of this dietary pattern on human health [45]. Reviewed literature also shows data on biological and agricultural biodiversity of the Mediterranean region, its food culture, and millennial cultural heritage, which is currently under threat due to food globalization and a shift toward unhealthy dietary patterns [46]. The four pillars of a proposed framework, including health, environment, economy, and society/culture, summarize the expression of the Mediterranean diet as identity and diversity of food cultures and systems across the Mediterranean region [47]. The key features leading to the lower environmental impact include the limited consumption of animal protein sources in favor of vegetable sources, such as legumes, that have also been associated with better health outcomes [48]. Also, the higher consumption of fruits and vegetables, together with whole grain cereals, would help to provide dietary antioxidants and fiber and a decreased risk of several non-communicable diseases [49]. On the downside, protein sources commonly consumed in the Mediterranean countries, especially by populations living in the islands as the one examined in the present study, are fish and seafood, which have been associated with various health benefits but also probably represent the greatest threat for certain environmental aspects (i.e., energy and freshwater eutrophication) when considering a global adoption of a Mediterranean-type diet. However, animal foods represent valuable dietary components of the Mediterranean diet, which should be considered within the context of sustainable food production. Besides fish and seafood, meat (red and poultry) may also be locally produced and have controlled procedures in order to maintain a low environmental impact and provide health benefits and offer health benefits not otherwise provided by plant products. Despite that not all sources of animal-food are equal, an ideal distribution of such foods may have a very different impact on the environment: thus, a low consumption of (unprocessed) meat or fish might be still in line with sustainable dietary choices, while exceeding in other foods (i.e., dairy products) may have detrimental impact less considered in other studies. These observations are in line with the results of our study: some individual food groups, for instance, cereals, may have an important environmental impact in Mediterranean countries because they are consumed in a higher quantity; moreover, in our study we found that consumption of meat was slightly higher among individuals with the eco-friendliest dietary habits, suggesting a general low consumption of meat (especially processed) in this population in spite of a high intake of dairy products. Interestingly, a study argued that while dietary patterns with all meat and dairy replaced with plant-based foods would lower environmental impacts, estimated intakes of zinc, thiamin, vitamins A, and B12, and probably calcium, would be below recommendations; thus, replacing 30% of the aforementioned food groups would result beneficial for saturated fatty acids, sodium, fiber, and vitamin D intakes, neutral for other nutrients, while reducing environmental impacts [50]. Notably, despite that there is agreement among current scientific literature on the topic, there are also other points of view to be considered when examining our results. In fact, it is questionable whether the Mediterranean diet was a more sustainable choice because the study was conducted on a population living in a Mediterranean area. A recent modeling study comparing a “healthy US-style” diet with a Mediterranean one pointed out that both dietary patterns would have a similar environmental impact except for freshwater eutrophication, which would be higher in the latter due to increased seafood consumption [51]. Notably, another modeling study investigating the ecological footprint in Mediterranean countries showed that the region’s ecological deficit can be further reduced by 8% to 10% [52]. 

The results presented in this study should be considered in light of some limitations. There is no univocal method used to assess the environmental impact of diet; thus, data generated are not quantitatively comparable. Dietary data are estimated through FFQs, which is not a perfect instrument to assess dietary intakes: they tend to overestimate food intake and may suffer from recall bias. Moreover, the development of the score is based on general estimates of the environmental impact of food production, but we cannot rule out the possibility that some food products may be locally produced and have different impacts on the domains investigated. However, these limitations are common to all studies sharing similar methodologies.

## 5. Conclusions

In conclusion, healthy dietary choices, including adherence to the Mediterranean diet, represent a desirable option nowadays when considering the environmental impact related to dietary habits of a population living in the Mediterranean area. Dietary changes are desirable, if not needed, to preserve both human and environmental health. It is of interest for the public health sector include incorporating principles of sustainability in dietary recommendations; however, only rarely the environmental impact is taken into account in national guidelines: when compared to actual average diets worldwide, nationally recommended diets in high-income nations would be associated with reductions in GHG, eutrophication, and land use, with an inverse trend when considering middle- and low-income nations [53]. A mutual action is needed in order to reach a significant improvement of global environmental sustainability [54]: from individual consumers to population choices, equitable distribution of resources and healthy food availability, encouragement (through policy and incentives) of more sustainable agricultural, farming, and fishery practices, and reduce food waste production at the food supply and consumer level [55]. This is a global approach that can be achieved by promoting consumption of local food production, maintenance of traditional culinary choices (when healthy), and preservation of the cultural heritage of populations [56]. Further research in this area is needed to investigate poorly explored aspects that might influence the overall impact markedly, not limited to food production but also including distribution and waste.

## Figures and Tables

**Table 1:** Background characteristics of the study population by sustainability score groups (*n* = 1806).

	Sustainability Score			
	Low (*n* = 566)	Medium (*n* = 524)	High (*n* = 626)	*p-Value*
***Age, n (%)***				0.534
18–39	235 (35.8)	183 (34.9)	208 (33.2)	
40–59	237 (36.1)	174 (33.2)	229 (36.6)	
>59	184 (28.0)	167 (31.9)	189 (30.2)	
***Sex, n (%)***				0.642
Male	268 (40.9)	226 (43.1)	270 (43.1)	
Female	388 (59.1)	298 (56.9)	356 (56.9)	
***BMI, n (%)***				0.135
<25 kg/m^2^	303 (50.1)	237 (49.1)	253 (43.0)	
25–30 kg/m^2^	203 (33.6)	163 (33.7)	226 (38.4)	
>30 kg/m^2^	99 (16.4)	83 (17.2)	110 (18.7)	
***Educational level, n (%)***				0.234
Low	218 (33.2)	198 (37.8)	233 (37.2)	
Medium	269 (41.0)	183 (34.9)	229 (36.6)	
High	169 (25.8)	143 (27.3)	164 (26.2)	
***Occupational level, n (%)***				0.579
Unemployed	165 (28.8)	119 (26.0)	157 (30.5)	
Low	87 (15.2)	80 (17.5)	73 (14.2)	
Medium	147 (25.7)	129 (28.2)	130 (25.3)	
High	173 (30.2)	130 (28.4)	154 (30.0)	
***Health status, n (%)***				
Cardiovascular disease	46 (7.2)	47 (9.1)	55 (9.0)	0.392
Cancer	28 (4.3)	15 (2.9)	32 (5.1)	0.160
Hypertension	336 (51.2)	278 (53.1)	308 (49.2)	0.426
Type-2 diabetes	43 (6.6)	45 (8.6)	55 (8.8)	0.267
Dyslipidemias	126 (19.2)	89 (17.0)	119 (19.0)	0.570
***Smoking status, n (%)***				0.148
Never	405 (61.7)	337 (64.3)	358 (57.2)	
Current	155 (23.6)	121 (23.1)	170 (27.2)	
Former	96 (14.6)	66 (12.6)	98 (15.7)	
***Physical activity, n (%)***				0.025
Low	107 (18.3)	84 (17.8)	120 (21.9)	
Medium	269 (45.9)	248 (52.7)	272 (49.7)	
High	210 (35.8)	139 (29.5)	155 (28.3)	

**Table 2:** Distribution of major food groups, micro- and macro-nutrient intake of the study population by sustainability score groups.

	Sustainability Score			
	Low (*n* = 566)	Medium (*n* = 524)	High (*n* = 626)	*Ptrend*
	*Mean (SD)*			
Energy (kcal/d)	2046.52 (718.62)	2080.21 (820.80)	2227.51 (866.94)	<0.001
***Macronutrients***				
Carbohydrates (g/d)	297.09 (115.91)	302.51 (128.94)	325.97 (132.65)	<0.001
Protein (g/d)	85.25 (31.97)	86.31 (36.52)	94.78 (44.15)	<0.001
Saturated total (% of energy)	24.15 (10.44)	24.29 (11.85)	25.42 (11.86)	0.045
Monounsaturated total (% of energy)	25.97 (9.57)	26.20 (11.49)	27.30 (11.48)	0.029
PolyunsaturatedTotal (% of energy)	11.24 (4.99)	11.19 (5.73)	12.12 (6.16)	0.006
Cholesterol (mg/d)	188.45 (92.30)	195.97 (106.46)	216.58 (128.93)	<0.001
Omega-3 (mg/d)	1.73 (0.93)	1.61 (0.77)	1.86 (1.01)	0.019
***Micronutrients and minerals***				
Vitamin A (Retinol eq, µg/d)	900.68 (467.31)	876.09 (424.48)	938.94 (519.10)	0.155
Vitamin C (mg/d)	160.99 (103.70)	161.30 (106.03)	180.60 (136.90)	0.003
Vitamin E (mg/d)	8.85 (3.50)	8.90 (4.03)	9.53 (4.52)	0.002
Vitamin D (µg/d)	5.20 (5.29)	5.36 (5.73)	7.01 (8.16)	<0.001
Vitamin B12 (µg/d)	6.21 (4.25)	6.66 (6.86)	7.99 (9.96)	<0.001
Sodium (mg/d)	2820.11 (1101.65)	2826.34 (1051.11)	2947.66 (1177.80)	0.046
Potassium (mg/d)	3666.22 (1359.90)	3566.88 (1188.16)	3808.60 (1473.28)	0.071
***Food groups (g/d)***				
Cereals	214.09 (127.26)	217.92 (146.97)	231.96 (138.74)	0.020
Fish	60.41 (60.64)	65.14 (76.02)	84.77 (105.84)	<0.001
Meat	69.18 (37.24)	70.30 (41.45)	74.62 (40.27)	0.014
Red Meat	32.91 (24.16)	34.40 (27.40)	36.14 (27.55)	0.029
Processed Meat	16.36 (18.86)	17.93 (21.75)	17.51 (23.07)	0.327
Dairy Product	207.24 (184.96)	195.98 (175.64)	188.83 (168.24)	0.062
Eggs	3.73 (6.14)	2.67 (5.06)	1.27 (2.31)	<0.001
Olive oil	7.30 (2.98)	7.33 (3.13)	6.92 (3.16)	0.032
Fruit	388.80 (310.41)	391.44 (307.84)	455.95 (390.49)	0.000
Vegetable	277.23 (170.72)	271.03 (173.04)	284.03 (220.62)	0.530
Legumes	36.74 (39.35)	36.63 (48.23)	45.43 (65.01)	0.003
Nuts	22.67 (36.09)	21.55 (42.56)	24.04 (50.29)	0.578
Fiber	32.23 (15.45)	32.42 (17.43)	36.11 (20.92)	<0.001
Alcohol	7.03 (11.47)	7.96 (12.07)	7.58 (11.94)	0.398

**Table 3:** Linear association of various dietary patterns index scores with individual components and total sustainability score.

	Land Use	Water Use	Energy	GHG	Sustainability Score
	*Beta (SE)*				
***Mediterranean diet***	−0.016 (0.013)	−6.408 (5.664)	−0.042 (0.032)	−0.012 (0.006) *	0.009 (0.004) *
***DASH***	−0.006 (0.048)	8.089 (21.702)	0.138 (0.122)	0.010 (0.024)	0.016 (0.016)
***Nordic diet***	−0.048 (0.020) *	−19.283 (8.820) *	−0.087 (0.050)	−0.018 (0.010)	0.018 (0.007) *
***DQI-I***	−0.016 (0.008) *	−5.831 (3.534)	−0.021 (0.020)	−0.005 (0.004)	0.006 (0.003) *
***AHEI***	−0.012 (0.007)	−5.939 (3.345)	−0.031 (0.019)	−0.008 (0.004) *	0.005 (0.003) *

* *p* < 0.05. AHEI, Alternate Healthy Eating Index; DQI-I, Diet Quality Index International; DASH, Dietary Approach to Stop Hypertension; SE, standard error.

## Supplementary Materials

The following are available online at https://www.mdpi.com/1660-4601/17/5/1468/s1.
