# Supplementary material for: Environmental Impact of Dietary Choices: Role of the Mediterranean and Other Dietary Patterns in an Italian Cohort
Source: Int J Environ Res Public Health. 2020 Feb 25;17(5):1468. doi: 10.3390/ijerph17051468 (PMC7084186; doi:10.3390/ijerph17051468)
Supplement: Supplementary file 1 [file ijerph-17-01468-s001.pdf]

Supplemental: Main environmental footprint information sources for items´ production and processing phases

LAND USE:

- Food and Agriculture Organization of the United Nations: data base 2009 FAOSTAT

Available: www.faostat.fao.org/ [accessed September 2016].

- Cederberg C & Mattsson B (2000) Lifecycle assessment of milk production – a comparison

of conventional and organic farming. *Journal of Cleaner Production* **8**, 49-60.

- Nielsen PH, Nielsen AM, Weidema BP *et al.* (2003) LCA food data base. Available:

www.lcafood.dk [accessed September 2016].

WATER CONSUMPTION:

- Herldbo J (2014) Recirculated aquaculture systems. Advantages and disadvantages. Good

Practice Workshop, Copenhagen, Denmark.

Available: https://circabc.europa.eu/sd/a/6112e063-d8aa-4533-9fbb-

2abd47cce769/Presentation%204%20Jesper%20Heldbo%20EU_Baltic_Recirculated%20Aquaculture_

JH.pdf [accessed September 2016].

- Hoekstra AY (2008) Water footprint of food. Available:

http://waterfootprint.org/media/downloads/Hoekstra-2008-WaterfootprintFood.pdf (accessed

September 2016).

- Hoekstra AY (2012) The hidden water resource use behind meat and dairy. *Animal frontiers*

2, 3–8. ISSN 2160-6056. Available: http://purl.utwente.nl/publications/81617 [accessed September

2016].

- Mekonnen MM & Hoekstra AY (2011) The green, blue and grey water footprint of crops

and derived crop products. Hydrology and Earth System Sciences. *Hydrol Earth Syst Sci* **15**,1577–

1600.

- Ministerio de Medio Ambiente y Medio Rural y Marino: (Ministry of environment, rural

and marine, Spain) (2006) Guía de Mejores Técnicas Disponibles en España del sector de Productos

del mar (Best Available Techniques Guide of Sea products sector in Spain). Spain. Available: http://www.prtres.es/data/images/Gu%C3%ADa%20MTD%20en%20Espa%C3%B1a%20del%20sector%20de%20productos%20del%20mar-3D0CDD9B58C62B31.pdf [accessed September 2016].

ENERGY CONSUMPTION:

- Carlsson-Kanyama A & Faist M. Energy use in the food sector: a data survey Available: http://citeseerx.ist.psu.edu/viewdoc/download?rep=rep1&type=pdf&doi=10.1.1.205.8375 [accessed September 2016].
- Foster C, Green K, Bleda M *et al.*(2006) Environmental Impacts of food production and consumption: A report to the Department for environment, food and rural affair. Manchester Business School. Defra, London. Available: http://www.ifr.ac.uk/waste/Reports/DEFRA-Environmental%20Impacts%20of%20Food%20Production%20%20Consumption.pdf [accessed September 2016].
- Garrido A, Bardají I, De Blas C *et al.* (2011) Indicadores de sostenibilidad de la agricultura y ganadería españolas (Spanish agriculture and livestock indicators of sustainability). Available: http://www.eurocarne.com/daal?a1=informes&a2=Informe_final_7868.pdf [accessed September 2016].
- Gołaszewski J, de Visser CLM, Brodziński Z *et al.* (2012) State of the Art on Energy Efficiency in Agriculture. Country data on energy consumption in different agroproduction sectors in the European countries. Available: http://www.agree.aua.gr/files/agree_state.pdf [accessed September 2016].
- Hambly, J (2011) Environmental – Ecological Impact of the Dairy Sector (Literature Review on Dairy Products for an Inventory of Key Issues – List of Environmental Initiatives and Influences on the Dairy Sector). *International Journal of Dairy Technology* **64**, 145–146.

- Hornborg S, Ziegler F (2014) Aquaculture and energy use: a desk-top study. Available: http://vbcv.science.gu.se/digitalAssets/1536/1536133_publication---energy-use-in-aquaculture.pdf [accessed September 2016].
- Masanet E, Therkelsen P, Worrell E (2012) Energy Efficiency Improvement and Cost Saving Opportunities for the Baking Industry. An ENERGY STAR® Guide for Plant and Energy Managers. Available: https://www.energystar.gov/sites/default/files/buildings/tools/Baking_Guide.pdf [accessed September 2016].

GHG EMISSION:

- CleanMetrics Corporation. Food Carbon emission calculator (2011) Available: http://www.foodemissions.com/foodemissions/Calculator.aspx [accessed September 2016].
- Nielsen PH, Nielsen AM, Weidema BP *et al.* (2003). LCA food data base. Available: www.lcafood.dk [accessed September 2016].
